# Optimising the validity and completion of adherence diaries: a multiple case study and randomised crossover trial

**DOI:** 10.1186/s13063-016-1615-7

**Published:** 2016-10-10

**Authors:** Rachael Frost, Doreen McClurg, Marian Brady, Brian Williams

**Affiliations:** 1 NMAHP-RU, Glasgow Caledonian University, Glasgow, UK; 2 School of Health and Social Care, Edinburgh Napier University, Edinburgh, UK

## Abstract

**Background:**

Diaries are the most commonly used adherence measurement method in home-based rehabilitation trials, yet their completion and validity varies widely between trials. We aimed to: (1) generate theory to explain this variation, (2) create an optimised diary and (3) evaluate the optimised diary’s validity.

**Methods:**

*Stage 1. Development*: using a multiple case study approach, we collected trialist interviews (*n* = 7), trial publications (*n* = 16) and diaries (*n* = 7) from seven purposively sampled UK rehabilitation trials. We explored return rates, diary designs and trialists’ ideas as to what affected diary completion and validity. Using explanatory case study analysis, we developed a diary optimisation model. *Stage 2. Evaluation*: we compared a diary optimised according to several model components to one nonoptimised according to the same components in a randomised AB/BA crossover trial. Healthy adults aged 60+ years without mobility impairments undertook a home-based 8-week walking programme. They recorded walking duration and frequency for 4 weeks per diary. We hypothesised that the optimised diary would possess greater validity for self-reported adherence to walking duration (criterion: the Activpal accelerometer), assessed during each diary’s final week. Participants were blinded to the hypothesis. Secondary outcomes included test-retest reliability and acceptability. Ethical approval was granted from Glasgow Caledonian University.

**Results:**

Thirty-two out of 33 participants completed the study. Diaries did not significantly differ in validity, reliability or acceptability. Both diaries agreed closely with the Activpal when assessing duration adherence at a group level, however, inter and intraindividual variation in validity was high (mean difference (95 % limits of agreement (LOA): limits of agreement plot the difference between measurements collected using two different methods against their mean and thus assess the extent to which the two measures agree with each other)) optimised diary = 3.09 % (−103.3 to 109.5 %), nonoptimised diary = −0.34 % (−131.1 to 130.5 %), *p* = 0.732). We found similarly wide LOA for percentage of days adhered to and percentage of walks taken, whilst frequency adherence was underestimated. Participants rated both diaries as low-burden and equal numbers favoured each diary or were neutral. Preference appeared to impact minimally upon validity.

**Conclusion:**

Group-level adherence diary data are likely to be valid. However, individual diary data lack validity, which raises concerns if using this data in calculations such as predicting functional outcomes. Different diary designs are likely interchangeable, though unanticipated high variation meant that this study was underpowered.

**Trial registration:**

The trial was not eligible for registration in a clinical trial database as diary measurement property outcomes, not clinical health outcomes of participants, were assessed.

**Electronic supplementary material:**

The online version of this article (doi:10.1186/s13063-016-1615-7) contains supplementary material, which is available to authorized users.

## Background

Adherence measurement in clinical trials is paramount to assess the extent to which the effectiveness of a particular intervention depends on the received intervention dose and to determine whether null results arise from suboptimal adherence or ineffectiveness. Adherence can be defined in general terms, such as the World Health Organisation definition – the extent to which a patient follows recommendations agreed with the provider [1] – or as components of the prescribed behaviour, e.g. adherence to frequency, intensity, duration and the type or accuracy of behaviour [2]. Adherence is vital where interventions contain unsupervised home-based therapeutic activities; however, measurement is difficult as observing these behaviours is usually infeasible. Currently, self-report questionnaires have little evidence to support their use [3, 4] and though some electronic methods are valid and reliable [5], they are costly and are mostly limited to walking activity. Previous systematic reviews have found adherence diaries to be one of the most commonly used adherence measures in unsupervised exercise-based rehabilitation, home-based rehabilitation and nonpharmacological self-management interventions [3, 6, 7]. Diaries are advantageous as they require only limited retrospection, can measure a wide range of behaviours in differing levels of detail and can display patterns of change over time. They are additionally both economical and simple to administer.

Despite their potential importance adherence diaries are vulnerable to two major problems: reduced validity from back- and forward-filling, social desirability and simple forgetfulness; and missing data arising from noncompletion and nonreturn [8, 9]. Our previous systematic review [5] found that adherence diaries had evidence for moderate to excellent validity and acceptability, suggesting that whilst they can be used well in some situations, this was not always the case. The reasons behind this were unclear. Qualitative and quantitative assessments of questionnaire return rates highlighted several potential factors that may apply to diaries, including participants’ opinions of the trial, personal factors, such as forgetfulness, prewarning participants about the questionnaires, question order, question content and monetary incentives [10–13].

However, despite their popularity there is little evidence to support optimal design or use of adherence diaries within a trial. A single, effective and acceptable diary would facilitate consistency and comparability of adherence measurement across rehabilitation trials, increase confidence in the quality of the data collected by therapists or researchers and maximise the amount of adherence data collected from patients. We therefore aimed to (1) generate theory to explain the variation in validity, completion and return of adherence diaries, (2) create an optimised diary based upon this theory and (3) evaluate the optimised diary’s validity against a nonoptimised diary.

## Methods

### Stage 1: Development

In order to learn lessons from past diary creation and use and develop theory to inform an optimised diary, we adopted a case study approach. Case studies offer an in-depth exploration of a phenomenon in its surrounding context [14]. They incorporate qualitative and quantitative methods and emphasise the role of the surrounding context. Case studies are consequently ideal to understand why practices or processes work in some situations but not others [14, 15]. To identify factors influencing the validity, completion and return of adherence diaries, we therefore used a multiple case study approach based upon Yin’s explanatory and exploratory methods. This relies on literal or theoretical replications of findings across cases to provide greater explanatory power than a single case [14].

### Sampling and data collection

We purposively sampled seven UK allied health professional rehabilitation trials as cases according to diary return rates, intervention type, trial size and diary design. Basic searches of the UK Clinical Research Network database were used to identify eligible clinical trials. Eligible trials were UK-based, completed within the last 5 years, contained a home-based rehabilitation intervention for adults, measured adherence using diaries and had available data regarding diary completion, return and/or validity. We intended to include one or more cases in which electronic diaries or apps were used, but we could not locate any trials matching these criteria. For each case we collected an example diary (*n* = 7), relevant trial publications (*n* = 16), conducted an interview with the trialists (*n* = 7) and any other relevant data volunteered (*n* = 8). Where available, we reviewed how a sample of anonymised diaries had been completed (*n* = 4). Informed consent was provided by the trialists interviewed.

Quantitative data (return rates, participant demographics and trial characteristics) were also extracted. Researcher interviews were transcribed by RF and all qualitative data thematically analysed in NVivo 10 [16]. Codes and categories were identified across individual data sources, whilst matrices were used to display major issues within cases which were compared across cases using pattern matching [14, 17]. Categories and issues were modelled and triangulated with quantitative data to produce an overall explanatory model. Rival explanations (e.g. all diary outcomes can be explained by general context effects) were tested and incorporated into the model where evidence was found. We reviewed the codes and cross-case models to increase the dependability of the findings. Member checking was undertaken with the trialists interviewed to assess credibility and to ensure sufficient anonymity. Ethical approval for the study was granted by the Glasgow Caledonian University School of Health and Life Sciences Ethics Subcommittee (ref PA13/58).

### Case study results

**Fig. 1:**
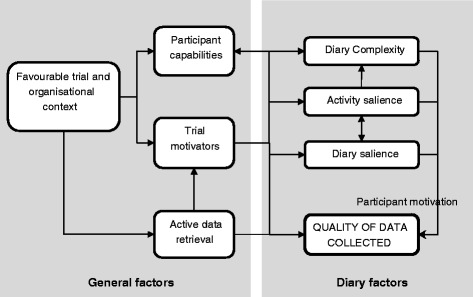
Model of factors influencing the quality of diary data collected

Diary return, completion and validity were summarised differently across cases and so qualitative classifications were used. Table 1 summarises each included case and its diary outcomes, Fig. 1 outlines the explanatory model developed and Table 2 explains each model factor. Note that ‘trialist’ refers to the trialist interviewed whilst ‘participant’ refers to those taking part in the trial studied.

**Table 1:** Summary of included cases

	Trial summary (setting)	Home-based activity measured (recording period)	Data collected	Diary description	Diary outcomes: Return Completion Validity
Case 1: NONSPEX	Single-blind feasibility study assessing the effects of including nonspeech oromotor exercises in SLT rehabilitation for persons with dysarthria following stroke (Home across 6 NHS health boards)	All participants (*n* = 39): Mins of words and sentence, conversation and (intervention only) nonspeech oromotor exercises per day and number of practices per day (7 weeks)	▪Interview ▪Sample diary ▪Viewed sample of completed diaries ▪Results publication [36] ▪Conference presentation [37]	3 A4 pages (×7); 126 items per week; frequency (tick), duration (number), comments (open); collected weekly	High – 100 % (32/32)
					Variable – a few people ‘physically not able to’ (NONSPEX: interview) complete diaries. A wide range of adherence was reported, suggesting variable completion. The random sample viewed in interview had variable completion.
					Unclear: ‘the therapist has recorded “some participants may have recorded more practice than they had carried out, some may have recorded less” (NONSPEX: interview)
Case 2: SCORD	Substantive pragmatic open trial assessing the use of night resting thermoplastic splints following hand surgery and therapy for Dupuytren’s contracture (5 NHS trusts, home)	Experimental (*n* = 77 + per-protocol deviations): Number of nights per week the splint was worn (6 months)	▪Interview ▪Sample diary ▪Viewed sample of completed diaries ▪Results, protocol and survey publications [38–40] ▪Relevant participant information sheet section	1 A4 page (×2); 3 items per week (completed weekly); frequency (number), comments (open); collected trimonthly	High – diaries returned completed in almost all cases (SCORD: telephone discussion). Successfully classified participants for a per-protocol analysis.
					High: ‘there might be [missing data]… I did dig out a couple of diaries, just very randomly … none of those had any missing lines’ (SCORD: interview). Random sample viewed in interview were complete.
					Unclear: ‘diaries were collected by research associates (not the treating therapists or surgeon) and, therefore, encouraged patients to be honest, independent verification of actual splint wear was not possible’ (SCORD: results publication). ‘What came back in the diaries was good, but it wasn’t too good to be true.’ (SCORD: interview)
Case 3: ENVISAGE-WP2	Pilot open trial (*n* = 22) comparing exergames, visualisations and exercise booklet only for secondary falls prevention (home only, single area)	All participants (*n* = 22): Number of individual exercises and exercise sessions undertaken (12 weeks)	▪Interview ▪Sample diary ▪Viewed sample of completed diaries ▪Results and protocol publications [41, 42] ▪Graph comparing computer and diary-recorded adherence	1 A4 page (×12); 0–70 items per week; frequency (tick); collected at end of study	High – 100 % (22/22)
					High: ‘there was a lot of detail in the diaries’ (ENVISAGE: interview). Sample of diaries viewed completed well.
					High: in the exergame group (*n* = 7) 7 weeks were correct, 5 weeks varied by <3/27 exercises across all participants.
Case 4: SELF pilot	Pilot pragmatic open trial comparing self-managed loaded PT exercise with usual PT for rotator cuff tendinopathy (single centre, private clinic)	Experimental (*n* = 12): Completion of sets of exercises per day (1 tick per session for 2 sessions a day) (3 months)	▪Interview ▪Sample diary ▪Viewed sample of completed diaries ▪Pilot results publication [43] ▪In -press results of substantive study ▪Substantive study protocol [44] ▪Qualitative pilot publication [45] ▪Patient and public involvement publication [46]	1 A4 page (×1–5); 7 items per week; frequency (tick); returned at each appointment	High: 92 % (11/12)
					Medium-high: 7 complete data (all consecutive diaries), 4 partial data. Returned diaries were well-completed on viewing, with the occasional incorrect completion. ‘reasonable degree of consistency’ (SELF: interview).
					Unclear: ‘therapists were very sceptical about this … you can see that sometimes the form and the pen is exactly the same and they’ve obviously been done quickly’. (SELF: interview)
Case 4: SELF substantive	Pragmatic open substantive trial comparing self-managed loaded PT exercise with usual PT for rotator cuff tendinopathy (3 NHS PT departments, home)	Experimental (*n* = 42): Completion of sets of exercises per day (one tick per session for two sessions a day) (3 months)		1 A4 page (×1–5); 7 items per week; frequency (tick); returned at each appointment	Low – 29 % (12/42)
					Medium: 5 complete data (all consecutive diaries), 7 partial data, ‘reasonable degree of consistency’ (SELF: interview) with occasional incorrect completion.
					Unclear: ‘maybe it’s not a 100 % accurate’ (SELF: interview).
Case 5: SUPER	Single-blind feasibility trial comparing PFMT and lifestyle advice after surgery for pelvic organ prolapse to advice leaflet only (3 NHS PT centres, home)	Experimental (*n* = 28): Number of sets of PFMT undertaken per day (12 weeks)	▪Interview ▪Sample diary ▪Results publication [47] ▪Participant information sheet	1 A5 booklet (folded A4) (×5); 21 items per week; frequency (number), comments (open); returned at each appointment	Low – 29 % (8/28) ‘about half of those I think that returned all five diaries all completed’ (SUPER: interview).
					Variable: ‘some of them are completed very well and very thoroughly and others are quite hard to understand really in what they actually meant’ (SUPER: interview).
					Unclear: ‘some of them were … pretty accurate and pretty well filled in’ (SUPER: interview).
Case 6: EVIDEM-E	Single-blind pragmatic substantive trial comparing an individually tailored progressive walking regimen with exercise therapist support to usual care in patients with behavioural and psychological symptoms of dementia (Home/community in several inner city, urban and semirural locations)	All participants (*n* = 131 patient-carer dyads): number and duration of walks per day plus qualitative walk data. Intensity (rating of perceived exertion) for experimental group only (12 weeks)	▪Interview ▪Sample diary ▪Results publication [48] ▪Protocol publication [49] ▪Viewing of anonymised diary dataset ▪Participant information sheet ▪Trial website	49 page A5 booklet (×1); 64 items per week; frequency (yes/no, number), duration (number), intensity (Borg scale – circle response), comments (open), whether completed set course (Yes/No), Why did not go out (circle response); collected at end of study	Medium: 68.7 % overall, intervention: 77.6 % (52/67), control: 59.4 % (38/64).
					Variable: observed diary database and some well-completed, some little to no data.
					Unclear: ‘I don’t think it’s reliable at all’ (EVIDEM-E: interview).
Case 7: TOMAS	Single-blind substantive trial (*n* = 568) comparing verbal advice, local travel information and PT/OT rehabilitation to verbal advice and travel leaflets only to improve stroke survivor outdoor mobility (Home/community in 15 NHS stroke services throughout Scotland, England and Wales)	All participants: number of journeys made per day (12 months)	▪Interview ▪Sample diary ▪Results publication [50] ▪Protocol publication [51] ▪Conference presentation relating to diaries [52] ▪Diary data coding sheet	12–13 page A5 booklet (×1); 14 items per week; frequency (number), falls (circle f); posted back monthly	Medium–high: 70.6 % of all expected travel diaries received and assigned. 89.4 % (508/568) returned at least one diary.
					Medium: 55.1 % returned all diary pages for 12 months. ‘Most of them you could use the data from them’ (TOMAS: interview). Extensive variations in recording required development of a data entry coding sheet.
					Unclear: ‘I imagine there was some people who didn’t keep them as accurate’ (TOMAS: interview).

*OT* occupational therapist, *PFMT* pelvic floor muscle training, *PT* physiotherapist, *SLT* speech and language therapist

**Table 2:** Supporting evidence for model factors

Factor	Subcategories	Examples and evidence	Influence
Favourable organisational and trial context Optimal organisational and trial contexts that produces a cost-effective, rigorous trial that is easily implemented	➢ Trial setting ➢ Organisational complexity ➢ Scientific rigour ➢ Practical concerns	‘In the NHS study what we saw was more loss to follow-up, more patients not attending treatment.’ (SELF: interview). ‘If money was no object, I’d’ve had someone ring them up every month to get them to send [the diaries] back’ (TOMAS: interview).	• Inhibited or facilitated the use of other factors. • If unfavourable, this became the researchers’ primary priority
Trial motivators Tangible and intangible benefits participants received through trial participation (e.g. greater clinical attention)	➢ Initial motivation for participation ➢ Treatment or other effects experienced ➢ Contextual effects (e.g. Hawthorne effect) of trial participation	‘[Control participants’] primary reason … for adhering to the exercise programme … was that they were taking part in a research study and had to record the sessions in a diary’ (ENVISAGE-WP2: trial publication). SCORD had high retention (96 %), high satisfaction (90 % for researcher phone/email contact and 91 % for visits) and all diaries were returned	• A net beneficial effect of participating appeared to generate a favourable attitude towards the trial, encouraging participants to complete and return all data required
Diary salience The extent to which the diary was emphasised to participants as a key data collection tool	➢ Initial diary status ➢ Positive and negative effects of diary completion ➢ Ongoing en/discouragement for diary completion	‘The therapist would always try and make sure it was hung up somewhere or out next to the, out next to the telephone or somewhere obvious’ (TOMAS: interview). ‘Something that we have to be careful with diaries on emphasising too much …they either you know lie if you like on the diary, or they feel that guilty they haven’t done it they don’t actually come back and see you’ (SUPER: interview).	• Increased participants’ effort to complete it well • Increased likelihood that participants would remember to complete it
Activity salience Distinctiveness of the behaviour carried out by the participants	➢ Actual adherence levels ➢ Integration of the behaviour into daily life	‘You wouldn’t really come back with a diary that said you only had done one set of exercises … I don’t know the patient would come in with that you know’ (SUPER: interview). ‘Some people said that it was very difficult to quantify the amount of conversation, because people would say things like “Och, I talk all day!”’ (NONSPEX: interview)	• A distinctive or frequent behaviour would be remembered more easily • More adherent participants would want to demonstrate this
Diary complexity The complexity of the diary design and the information it asked for	➢ Participant perceived burden ➢ Participant actual burden	‘Just to make it as simple as possible … we just wanted to reduce that cognitive load … you just tick whatever exercise you did and that was it, because that’s, that’s really all we were interested in anyway’ (ENVISAGE: interview). ‘[We thought] yep, that’s a 1-pager, simple enough, it fits 3 months’ worth of weekly recording’ (SCORD: interview).	• A more complex and difficult diary would provide more barriers to completion and reduce motivation
Participant capabilities Participant-related factors that influenced the level of diary data collected	➢ Participant impairments ➢ Other demands placed on participants (e.g. employment, social demands, caring)	‘There were a few people who hadn’t filled in the diary because they were physically not able to do it’ (NONSPEX: interview). ‘The carers were the main players in this, completing these diaries … most of them were working full time or caring for grandchildren, children’ (EVIDEM-E: interview).	• Impairments reduced ability to remember and record the exercise • Other demands reduced diary salience in light of other priorities
Active data retrieval Strategies used by trials to circumvent the need for participants to be motivated to complete or return the diaries	➢ Collection from participants’ homes ➢ Retrospective missing data completion with a therapist or researcher ➢ Completion assistance at appointments	‘It made it very easy for the patients. And to some extent we came to get the data from them…We weren’t waiting for responses by post’ (SCORD: interview). ‘We set it up that she would collect them on a week-by-week basis. You know we never considered any alternative to that as being er, as being viable’ (NONSPEX: interview).	• These strategies generally achieved high return rates and more complete diary data

Briefly, this multiple case study suggested that in order to collect high-quality diary data, the trial and organisational context first needed to be favourable. Trials experiencing problems at certain sites (e.g. due to physiotherapist illness) or which faced issues with recruiting and retaining sufficient participants, understandably focussed on addressing these issues rather than ensuring that the completion of adherence diaries was high. Secondly, trial motivators needed to be present. Trials in which patients experienced some benefit from participation, such as enjoyment of the trial visits or the opportunity to play exercise games (e.g. SCORD, ENVISAGE-WP2), tended to have higher overall trial engagement and, in parallel to this, higher diary return rates. Participants’ capabilities, such as cognitive or motor impairments or competing life demands, e.g. caring responsibilities, were also theorised to influence diary validity and return, though exploration of this factor was limited due to a lack of participant input.

When these general factors were optimal, three diary-related factors appeared to influence completion and validity. Perceptions of the diary as an important motivational or data collection tool (diary salience) appeared to increase return and completion, and this was increased through emphasis by therapists and researchers. The ease of recalling the activity (activity salience) seemed to improve validity and completion. Those with greater adherence were thought by trialists to be keener to demonstrate this in diaries and more distinctive behaviours appeared to be more easily recalled and recorded. Finally, the apparent visual complexity of the diary and the actual complexity (the type and amount of data they were required to complete) appeared to decrease completion and return rates.

Active data retrieval (direct strategies to retrieve diary data, e.g. collection from participants’ homes or therapist assistance with completion) further improved return and completion rates as they circumvented the need for participants to be motivated, though did not necessarily improve the validity of the data collected.

### Stage 2: Evaluation

**Fig. 2:**
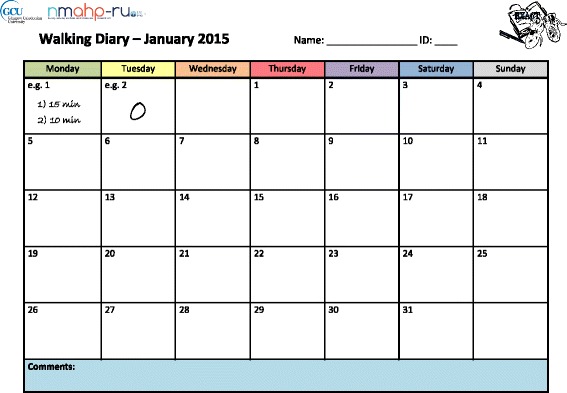
Optimised diary page

**Fig. 3:**
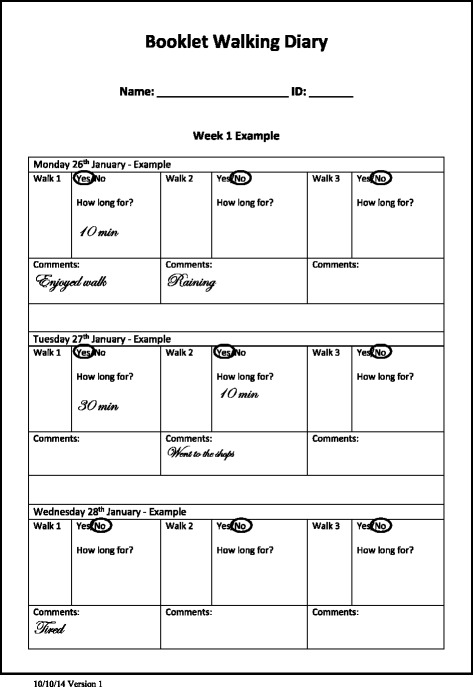
Example of a page from the nonoptimised diary

The above model contained a number of factors that could be optimised and tested. However, changes to the format and design of the diary were both economical and the most easily implementable in future research and practice. We based these changes upon the concepts of salience (a diary that engaged participants would be better completed) and complexity (a diary which collected fewer, simpler items spread across fewer pages would be better completed) identified in the case study model. As no ‘usual diary’ currently exists, we developed a package of design changes that would theoretically optimise one diary and compared this to a diary nonoptimised according to the same principles (Table 3, Figs. 2 and 3). Both diaries are also attached in Additional file 1. Our null hypothesis was that there would be no difference in the criterion validity of diaries when recording percentage adherence to daily walking duration.

**Table 3:** Differences between the optimised and nonoptimised diary

Conceptual basis	Diary element	Optimised diary	Nonoptimised diary
Diary complexity	Data per page	1 month	3–4 days
	Length	1–2 × A4 page	12 × A4 pages
	Format	Single sheet	Stapled booklet
	Spaces per day	1	6
	Type of data collected	Numerical	Yes/No
			Numerical
			Text (comments)
	Information required if have not walked	Single zero	Circling 3 ‘No’s
Diary salience	User-friendliness of design	Simple clean lines	Excessive lines and text
		No unnecessary text	Complex grid
		Font size 14	No accents for key information
		Study logos	Font size 11
		Takes up less space	No logo or image
		Displays weekly progress	Booklet hinders ability to see progress over weeks

We used a randomised AB/BA 1:1 crossover trial design. Validity was considered to be a fairly stable concept unlikely to be permanently affected by diary type, so we used a crossover design as it eliminates between-participant variation, giving greater statistical precision and requiring fewer resources. This further allowed us to directly compare the acceptability of the two diaries.

### Participants

Inclusion criteria were: healthy adults aged 60+ years, self-reported ability to walk for longer than 10 min unassisted and able to consent. Exclusion criteria (self-reported) were hip or lower leg problems impeding mobility; heart conditions; fall or major health problem within the last 6 months; visual impairment prohibiting reading the information sheet, diary or consent form; physical or motor impairments preventing basic writing; and people unable to speak, read or write in English at a basic level. Adults aged over 60 years were considered likely to use a rehabilitation measure in the future and this avoided limiting the findings to a sample with a single condition. Participants were recruited from the community in Hertfordshire, UK and were visited at home by RF. Informed consent was obtained from all participants.

Participants were given a written walking programme to carry out at home starting at 20 min/day and increasing by 5 min/day each fortnight for 8 weeks. We randomised participants to complete one diary for 4 weeks, immediately followed by the other diary (AB/BA), in which they recorded each walk taken per day and its duration in minutes. Ethical approval was granted from Glasgow Caledonian University School of Health and Life Sciences Ethics Subcommittee (ref HLS/Psy/A14/009).

### Outcomes

The primary outcome was the difference in criterion validity between the optimised and nonoptimised diary for assessing percentage adherence to daily walking duration. The ‘gold standard’ used was the Activpal, an accelerometer which attaches to the thigh using a waterproof dressing and detects time spent standing, stepping or sitting according to the inclination of the thigh [18]. It has good validity in older adults [19]. The Activpal can be worn continuously for a week and does not display feedback to participants. To prevent carryover between interventions, a common problem in crossover trials [20], the Activpal was worn for the final week of each 4-week period. The difference in percentage adherence to walking duration per day between the two methods was compared and averaged over the week.

Secondary outcomes included the difference in criterion validity between percentage adherence to walking frequency per week and percentage of days adhered to; differences in test-retest reliability for the same outcomes, compared between weeks 3 and 4 for each diary; and diary acceptability, assessed through percentage of days completed and a nine-item self-developed questionnaire, using visual analogue scales to assess burden and usefulness (Additional file 2). Semistructured interviews were carried out with participants purposively sampled according to validity, walking level, age and gender to further explore diary acceptability, explain the study results and refine the model from the case study.

### Sample size and randomisation

As data for a sample size calculation were lacking, we established a number of initial assumptions and tested these in an internal pilot (*n* = 10). Assuming 80 % power, alpha of 0.05 (two-tailed) and standard deviation (SD) of 20 %, we aimed to recruit 30 participants to detect a 15 % difference in validity. We decided through consensus that an arbitrary difference of 15 % in adherence would be the minimum to detect in a trial aiming to improve adherence and so interchangeable diaries would need to be within this threshold. However, the internal pilot found high variability (SD = 51.2 %), requiring an infeasibly large number (*n* = 184) of participants within the time and resources available. We therefore halted recruitment at 33.

We used Randomization.com [21] to block randomise (block size 10) participants to each group. Sequence generation was undertaken by a colleague (SL) and concealed from the chief investigator (RF), who screened and enrolled participants, until 2 days prior to the first appointment. RF was the sole investigator and so could not be blinded at outcome assessment. However, outcomes were self-reported and participants were blinded to the hypothesis that one diary had greater validity (diaries were referred to as ‘Calendar’ or ‘Booklet’).

### Analysis

Data were input into Excel for preliminary calculations and exported to SPSS 21 for further analysis. Dual data extraction was undertaken for a random 10 % of all data and met the minimum planned criteria of over 90 % agreement. Participants were included in the validity analysis if they had at least 4 days’ Activpal data. Where days were missing from the Activpal, the matching day was excluded from the diary in validity calculations. Missing diary data were assumed to be zero. Activpal walking bouts were identified using an Excel programme tailored to the study according to the following parameters: total walking time at least 10 min, no pauses for 60 s or longer and an overall cadence of 60–120 steps/min. These cutpoints were developed prior to the analysis based on previous literature [22, 23] and which best matched the graphical Activpal output.

For validity outcomes, we calculated the mean differences between the diary and Activpal and plotted the limits of agreement using Bland-Altman plots [24]. We used regression modelling, with day (duration only), period and allocation as random effects and participant (within allocation) as fixed effects, to test the paired differences between each outcome [20]. Significance was set at *p* = 0.05. Where paired differences were not normally distributed for an outcome, we used period-adjusted Mann-Whitney-Wilcoxon tests or sign tests. Reliability analyses were undertaken in the same way between the third and fourth weeks for each diary and Pearson correlations calculated. Acceptability questionnaire data were visually plotted and compared using the approach above. We made the a priori decision to adjust all outcomes for period effects. Period effects are potential systematic differences between the two periods in the crossover design, e.g. participants becoming habituated to recording walking over time, which could potentially increase the validity of estimates in period 2. We designed the study to prevent carryover as recommended by Senn [20] and tested for this to confirm our assumptions. We used framework analysis [25] to analyse qualitative interview data. Figure 1’s model was the guiding framework and categories were refined or newly developed as needed.

## Results

**Fig. 4:**
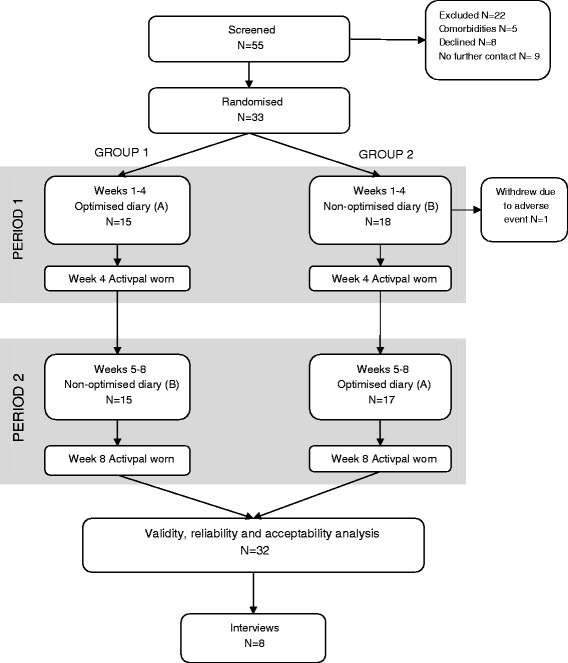
Flow of participants throughout the study

Thirty-three individuals were recruited between December 2014 and March 2015 and 32 completed the study and were analysed (Fig. 4). One participant withdrew due to back pain developed during a long walk in the first week of the study. Participants were largely in their 60s, female and highly educated (Table 4). Adverse events (*n* = 7 events in *n* = 5 participants) were mild and were related to Activpal dressings (e.g. local redness or itching).

**Table 4:** Demographics of participants completing the study

	Group 1 (*n* = 15)	Group 2 (*n* = 17)	Total (*n* = 32)
Mean (SD) age	66.6 (4.56)	69.9 (6.57)	68.3 (5.87)
Gender (M/F)	4/11	8/9	12/20
Level of education (%)			
Higher education & equivalents	12 (80 %)	14 (82 %)	26 (81 %)
A levels and equivalents^a^	0 (0 %)	0 (0 %)	0 (0 %)
Apprenticeships, GCSEs and equivalents^b^	1 (7 %)	1 (6 %)	2 (6 %)
Level 1 and below	0 (0 %)	0 (0 %)	0 (0 %)
No qualifications	2 (13 %)	2 (12 %)	4 (13 %)

^a^School- or college-leaving qualifications usually taken around age 18

^b^School-leaving qualifications usually taken around age 16

All participants completing the study had at least 4 days of Activpal recording. Activpal data loss occurred from low battery (*n* = 1, 3 days; *n* = 1, 2 days; *n* = 1, 1 day) and from early removal due to skin irritation (*n* = 2, 1 day). Corresponding diary data for these days were removed from the validity analysis. Not all participants completed the diary for 28 days due to logistical issues (optimised diary, *n* = 4, 27 days; nonoptimised diary, *n* = 1, 27 days; *n* = 1, 26 days; *n* = 1, 25 days). These days were from the start of the diary (completion analysis only) and were unrelated to allocation. Two participants had a 3-week gap between periods due to bereavement or forgetting the (optimised) diary.

Participants walked a relatively consistent amount throughout the study (55.2 min (range 9.3 to 175.7 min) per day in week 1 and 63.3 min (range 8.6 to 187.9 min) in week 8). Walking frequency averaged 9.9 (3 to 25) in week 1 and 10.8 (1 to 31) per week in week 8. Participants appeared to prefer to set their own consistent walking targets rather than followed the prescribed increasing targets – the number of days adhered to decreased from 4.8 to 3.8 throughout the study as the recommended duration increased.

### Outcomes

**Fig. 5:**
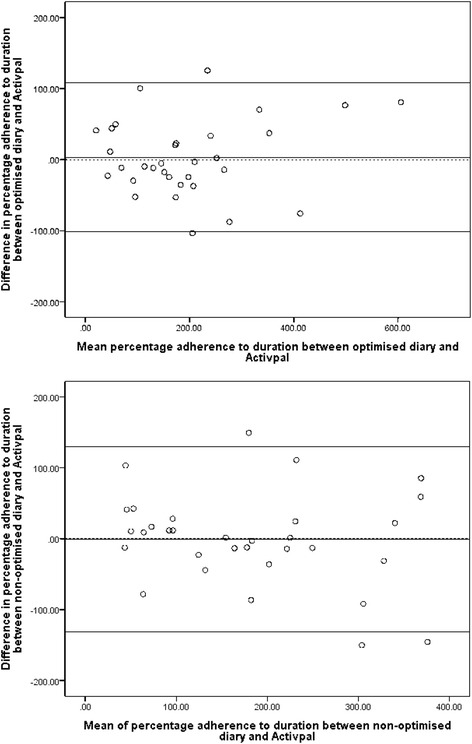
Bland-Altman plots: criterion validity of optimised (top) and nonoptimised (bottom) diary compared to the Activpal

Table 5 shows the validity and reliability outcomes. For the primary outcome, percentage adherence to walking duration, both diaries on average agreed with the Activpal (optimised = 3.09 %, nonoptimised = −0.34 %). This difference was not significant (3.44 %, *t*(401.8) = 0.342, *p* = 0.732) and the null hypothesis could not be rejected. Limits of agreement (LOA) showed large interindividual variation in participants’ validity (Fig. 5) (optimised diary 95 % LOA = −103.32 % to 109.50 %; nonoptimised diary 95 % LOA = −131.13 % to 130.45 %). LOA between the validity for each participant for each diary also varied widely (−101.2 % to 108.0 %), suggesting high intraindividual variation was also present.

**Table 5:** Validity and reliability outcomes

	Optimised diary	Nonoptimised diary	Difference
	Mean difference (95 % LOA)	Mean difference (95 % LOA)	Mean (*p* value)
Validity: percentage adherence to duration	3.09 % (−103.3 to 109.5)	−0.34 % (−131.1 to 130.5)	3.44 % (*p* = 0.732)
Validity: percentage adherence to frequency	−72.4 % (−282.5 to 138.7)	−64.0 % (−237.1 to 109.1)	−8.43 % (*p* = 0.672)
Validity: percentage of days adhered	2.94 % (−44.51 to 38.63)	1.05 % (−31.05 to 33.15)	−3.99 % (*p* = 1.00)
	Pearson correlations (mean difference, 95 % LOA)	Pearson correlations (mean difference, 95 % LOA)	Mean (*p* value)
Reliability: percentage adherence to duration	*r* = 0.702 (−13.5 %, −69.9 to 42.9)	*r* = 0.622 (−5.42 %,−60.4 to 49.5)	−8.06 % (*p* = 0.147)^a^
Reliability: percentage adherence to frequency	*r* = 0.740 (−29.4 %, −147.3 to 88.5)	*r* = 0.788 (−14.6 %, −103.5 to 74.4)	−14.8 % (*p* = 0.270)
Reliability: percentage of days adhered	*r* = 0.655 (−3.42 %, −50.0 to 43.2)	*r* = 0.652 (−3.17 %, −49.7 to 43.4)	−0.76 % (*p* = 0.899)

^a^Evidence of period effects (*p* = 0.006)

Similarly, no significant differences and wide intra and interindividual variation were found for other validity and reliability outcomes (Table 5, see Additional file 3 for individual Bland-Altman plots for each outcome). Narrower LOA were found for validity of the percentage of days adhered to per week, whilst walking frequency adherence was substantially lower in both diaries with wider LOA. Test-retest reliability analyses showed moderate to high correlations and narrower LOA than for validity. Period effects were present for test-retest reliability of walking duration, but for all other outcomes there was no evidence of period effects or carryover.

Acceptability was similar between diaries. Percentage of days completed did not differ between diaries as to whether any data were present per day (median = 100 % for both, sign test *p* = 0.378) or whether basic frequency and duration data were completed (median = 100 % for both, *W* = 266.0, *p* = 0.553). The percentage of days completed exactly as requested was significantly higher in the optimised diary (86.4 % versus 65.5 %, *t*(30) = 2.539, *p* = 0.017). Similar numbers of participants preferred the optimised diary (*n* = 12), the nonoptimised diary (*n* = 11) or were neutral (*n* = 9). The average preference recorded on the VAS (0 = optimised diary, 100 = nonoptimised diary) was 47.06 (SD 34.4). There was a slight tendency for participants to prefer the first diary they had completed, but this was not significant (post-hoc *t* test *p* = 0.379).

**Fig. 6:**
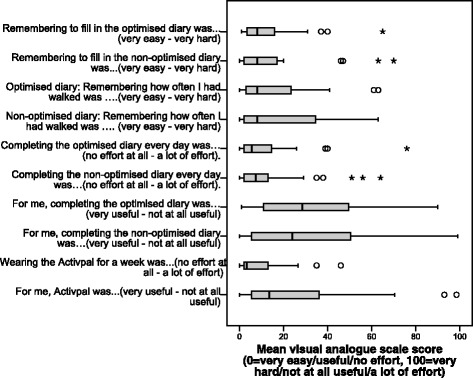
Acceptability scores for each diary (0 = very easy/useful/no effort, 100 = very hard/not at all useful/a lot of effort)

Figure 6 shows the mean values for other acceptability questionnaire outcomes. Overall, the acceptability questionnaire showed that both diaries were equally easy to use and presented only a low burden. Most participants completed the diaries daily (optimised, *n* = 21, 66 %; nonoptimised, *n* = 15, 53 %) or after every walk (optimised, *n* = 8, 25 %; nonoptimised, *n* = 11, 34 %), with no differences between the diaries (*W* = 278.00, *p* = 0.941). Small numbers completed the diaries every few days or once a week. The majority of participants took less than 2 min to complete an entry (median = 1 min for both, *W* = 213.50, *p* = 0.163).

### Post-hoc exploratory analyses

**Fig. 7:**
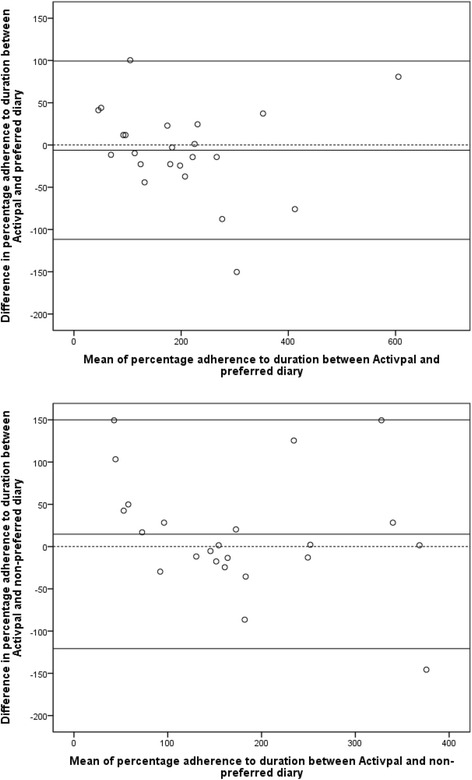
Bland-Altman plots for validity in preferred (top) and nonpreferred (bottom) diaries

In light of the unexpected findings, we used a small number of post-hoc analyses to further explore the data. As preference was divided across participants, we explored the effect of this (preferred versus nonpreferred diary, *n* = 23) upon duration adherence validity. Though narrower limits of agreement were found (preferred −6.2 % (LOA −112 % to 99.4 %), nonpreferred 14.6 % (LOA −121 % to 150 %) (Fig. 7), this difference was not significant (*p* = 0.179) and no differences were found for other validity outcomes. Using a scatter plot to individuals’ consistency in validity across diaries, we found that individuals were largely consistent as to whether they over or underestimated adherence, but the magnitude of this varied widely.

Finally, as there appeared to be no differences between the diaries, we pooled the data from both and assessed responsiveness to an increase in walking between weeks 4 and 8 detected by the Activpal (13.9 min, *t*(31) = −3.063, *p* = 0.005). Combined diary data found an increase of 14.0 min (*t*(31) = −2.698, *p* = 0.011), suggesting that the diaries were responsive to change.

### Qualitative interviews

We carried out eight semistructured interviews. Preference for diary formats varied between participants, though most interviewees considered the nonoptimised diary a bulky waste of paper. The optimised diary was considered simpler and easier but the reduced space annoyed participants who had large handwriting or wanted to make notes. However, these considerations did not appear to influence completion or preference between diaries:

‘they both have good points and bad points. One wasn’t easier to fill in than the other.’ (Participant #20)

Generally, the nonoptimised diary was preferred by participants with lower amounts of walking as this contained a comments box and so they could explain why they had not walked:

‘I could write that I’d been to yoga or I’d been doing something else and so I needn’t feel bad that I only did one walk.’ (Participant #13)

**Fig. 8:**
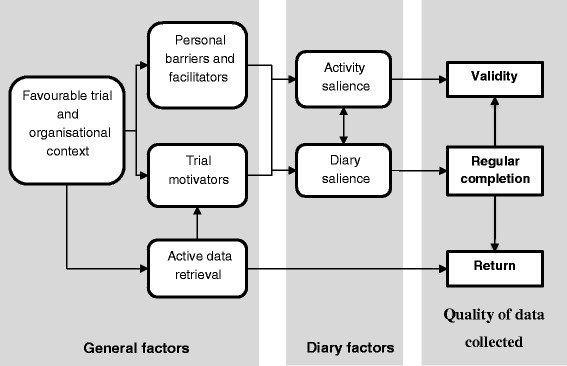
New model of factors influencing diary validity and completion

As the complexity of the diary appeared to vary with participants, the previous model developed was reiterated in light of the results (Fig. 8). The complexity category was subsumed into personal barriers and facilitators along with participant capabilities, as interviews revealed a multitude of personal factors, such as habits and interruptions to routine. These could not be explored in the case study as it was undertaken at a trial level, but appeared to contribute to regular diary completion, validity and walking:

‘This time, well what I call a timetable [the optimised diary] because it’s the kind of template that I’ve worked with all my life. It’s a bit like a teaching – you know I used to prepare timetables for my staff and this is what it looked like so this is very familiar to me.’ (Participant #26)

Most participants noted that leaving the diary out in a memorable place encouraged regular completion, supporting the concept of diary salience. However, participants did not see much personal benefit from completing the diaries and saw it as mainly beneficial for the research only:

‘So for me it was relatively easy in as much as the dining room table was fairly empty, it was sitting there, all I had to do was fill it in and I occasionally walked past and thought “must fill you in”.’ (Participant #10)

There was large support for the concept of activity salience, in that walking for pleasure or activity was better remembered by participants than walking for functional activities (e.g. shopping, going to the postbox), and those undertaking larger amounts of walking found it more difficult to recall precisely how long they had walked for:

‘Because, in my lifestyle all exercise in excess of 10 minutes, which was the goal, is manmade … So you remember every time that you’ve actually done something where you did consciously say I am going to go and walk.’ (Participant #21)

Both the diaries and the Activpal increased participants’ awareness of how much they walked, but there were mixed opinions as to how motivational diaries were. The Activpal was seen to be more motivational, partly as participants could only change the data recorded by walking more – within the diaries participants could compensate for their perceived low walking levels by extending the definition of walking:

‘I didn’t do proper walks did I? … I was counting things like going shopping, walking round the shops which I know is not really a good walk.’ (Participant #26)

There was also some support for trial motivators – participants mentioned that being in a trial was an added motivation to complete the diary, and some enjoyed personal benefits from the trial (e.g. Activpal feedback).

## Discussion

We used a multiple case study of seven UK home-based rehabilitation trials to develop a theoretical model to improve the validity, completion and return of adherence diaries. We tested two of the diary-related factors, diary complexity and salience, by designing an optimised and a nonoptimised walking adherence diary, completed in a randomised crossover trial by healthy older adults for 4 weeks each. The primary outcome was the criterion validity of percentage adherence to minutes of walking per day, assessed through comparison with an Activpal worn for the fourth week of each diary. Secondary outcomes included criterion validity of walking frequency data and percentage of days adhered to, test-retest reliability of these adherence outcomes and acceptability (percentage completion and self-developed questionnaire). No differences were found between the two diaries, though the study was underpowered. Both were, on average, valid, but individually possessed extreme variability. Analogous results were obtained across other outcomes, apart from underestimation of walking frequency and significantly higher completion exactly as requested in the optimised diary. Both diaries were similarly acceptable and easy to use.

These findings contrast some of the previous diary literature. Other studies have found that individuals over-report walking frequency and under-report walking duration [26], that equal numbers over- and under-report exercise session frequency [27] and that under-reporting occurs when people are aware that they are being monitored [28]. Our study did not find a trend towards under- or over-reporting for any outcome apart from frequency. However under-reporting of frequency was likely to reflect the Activpal cutpoints used, as longer walks tended to contain pauses of longer than 60 s and so were classified as two or more walks. This was necessary to accurately detect walking duration, but obscures the true estimate of frequency validity.

The extensive variability found in our study was supported by another small study of 11 African American women with systemic lupus erythematosus, where the limits of agreement between a diary and WiiFit were −27 to 35 min per session for a 30-min prescription [29]. It is, therefore, possible that high inter and intraindividual variation is prevalent within diaries but masked by the use of correlational statistics in some validity studies [30, 31]. There was clear evidence of digit preference for duration that may have further contributed to individual variability. Similarly to one other study [30], diaries appear to be reliable, though this property is not always considered necessary in diaries as they are designed to show patterns over time [32].

Unlike the questionnaire design literature, for which there is substantial evidence to support some design changes in improving return rates [10, 12, 13], we found no differences in preferences and diary outcomes. This may be due to the simpler nature of the data collected in diaries or the use of validity as a primary outcome rather than return rates. However, the study was underpowered to detect a difference. A post-hoc power calculation found that only a 38 % difference in validity would have been detected in this study, and so it is possible that small differences were not detected.

Seminal adherence literature often assumes that diaries are motivational [33–35]. We found mixed evidence for this – the feedback from the Activpals appeared to be more motivational as there was a significant increase in walking between weeks 4 and 8, during which the Activpal feedback was returned to participants. However, feedback and discussion of the diary data was kept to a minimum during this study – it is possible that further discussion may have increased engagement with the diaries and their motivational effects, as theorised in our case study.

This study offered a novel approach to evaluating adherence diaries. The case study developed a strong theoretical basis for diary improvement, with strategies to improve credibility built into the study. However, we could only find one trial which assessed diary validity and none which used electronic diaries, which limited the scope of the model. Additionally, we could not access trial participants’ views within this case study, which may be one reason the intervention was ineffective at improving diary validity.

The crossover trial design used was robust. Period effects were only apparent for one outcome and there was no evidence of carryover, though tests for these outcomes are generally underpowered [20]. We included acceptability, an underexplored dimension of adherence measurement, as an outcome. We did not use member checking as it seemed unlikely that members would confirm aspects such as compensation. However, prolonged engagement by RF with the participants over the course of the study added further credibility to the findings, though risked introducing an element of social desirability bias. The major limitation of this study was that walking was undertaken in healthy, well-educated adults as a health behaviour rather than as a therapeutic treatment. Motivations and concerns of participants may, therefore, differ somewhat from those undertaking rehabilitation, particularly as participants were not screened for low walking levels at baseline. However, some similarities to other studies [29] suggest the findings may apply to rehabilitation situations. It is further possible that participants made greater efforts to be valid as they were aware of being recorded [28], though qualitative evidence for this was mixed.

In comparison with other adherence measures, which currently lack good evidence of validity across nonpharmacological rehabilitation situations [3, 6, 7], this study offers the following implications for using diaries in research and practice:

- Diaries can be validly used where group-level adherence to activity duration is to be measured (e.g. group change, descriptive summaries) and where the activity is unambiguous, infrequent and easy to recall separately to other activities, e.g. a daily walking prescription for participants who do not often walk
- Diaries as they are currently designed should be avoided where individual-level comparisons are intended (e.g. as a predictor or outcome) or for functional, frequently performed behaviours which are more difficult for individuals to recall. Electronic measures or validated questionnaires may provide a better alternative to measure this. However, all measures still require further work and development before they can be validly used to assess adherence to complex regimens
- Advising participants to place the diary somewhere memorable and emphasising its importance appear to be key strategies to improve their completion within trials
- Researchers should focus on how easy activities are to recall and record when seeking service user input rather than design and complexity of diaries, which appears to have little impact on cognitively healthy participants
- Potentially more than one diary design could be used to collect the same data, according to patient preferences
- Diaries are likely to be influenced by how the trial is organised and carried out and the extent to which the trial and its context provides a net benefit for participants
- Clinicians should be aware that diary data is unlikely to be highly accurate for a given individual; nevertheless there is a lack of valid alternatives and diaries may offer motivational benefits for some patients

Further research is required to ensure these results apply to other populations, e.g. trial populations that are unwell, those currently in the active phase of rehabilitation and populations with mild cognitive impairment. Further adequately powered studies are required into the validity of diaries for recording adherence to complex rehabilitation activities, whilst electronic adherence diaries remain a valuable avenue for exploration. Simple strategies, such as placing the diary somewhere memorable or placing greater emphasis on the diary, require evaluation within the context of clinical trials.

## Conclusion

Adherence diaries remain a valuable method of adherence measurement when studying group adherence, when assessing activity duration and when an activity is easily defined by participants. However, they appear to lack validity on an individual level and so should be avoided when used to assess individual-level associations for predictors of adherence or outcomes. Clinicians should be aware that diary data is likely to vary highly in accuracy, though may provide motivational effects for some participants. Further confirmation of these findings is needed in a wider range of activities and populations.

## Additional files

Additional file 1: Optimised and nonoptimised diaries. (PDF 312 kb)
Additional file 2: EXACT: Acceptability Questionnaire. (PDF 273 kb)
Additional file 3: Bland-Altman plots for other analyses. (DOCX 192 kb)

## Abbreviations

LOA: Limits of agreement
OT: Occupational therapist
PT: Physiotherapist
PFMT: Pelvic floor muscle training
SD: Standard deviation
SLT: Speech and language therapist
