# Supplementary material for: Optimising the validity and completion of adherence diaries: a multiple case study and randomised crossover trial
Source: Trials. 2016 Oct 10;17:489. doi: 10.1186/s13063-016-1615-7 (PMC5057493; doi:10.1186/s13063-016-1615-7)
Supplement: Additional file 1: — Optimised and nonoptimised diaries. (PDF 312 kb) [file 13063_2016_1615_MOESM1_ESM.pdf]

Additional File 1: Optimised and non-optimised diaries

A. Optimised diary

Walking Diary – January 2015

Name: _________________ ID: ____

Monday	Tuesday	Wednesday	Thursday	Friday	Saturday	Sunday
e.g. 1<br>1) 15 min<br>2) 10 min	e.g. 2<br>0		1	2	3	4
5	6	7	8	9	10	11
12	13	14	15	16	17	18
19	20	21	22	23	24	25
26	27	28	29	30	31	

Comments:

Instructions

For each day, please write in the box the number of times you walked and how long each walk lasted.

Example 1

If you have walked twice today, once for 15 minutes and once for 10 minutes, you should write the following in today's box:

1) 15 mins
2) 10 mins

e.g. 1	e.g. 2
1) 15min<br>2) 10min	0

Example 2

If you have not walked for over 10 minutes at a time today, you should write 0 in the box.

If you have any comments, please write them in the blue Comments box at the bottom of the page.

Please try and remember to fill the calendar in daily. Thank you for participating in this study.

B. Non-optimised diary

Booklet Walking Diary

Name: ______________________ ID: _______

Week 1 Example

Monday 26th January - Example					
Walk 1	Yes/No<br>How long for?<br>10 min	Walk 2	Yes/No<br>How long for?	Walk 3	Yes/No<br>How long for?
Comments:<br>Enjoyed walk		Comments:<br>Raining		Comments:	

Tuesday 27th January - Example					
Walk 1	Yes/No<br>How long for?<br>30 min	Walk 2	Yes/No<br>How long for?<br>10 min	Walk 3	Yes/No<br>How long for?
Comments:		Comments:<br>Went to the shops		Comments:	

Wednesday 28th January - Example					
Walk 1	Yes/No<br>How long for?	Walk 2	Yes/No<br>How long for?	Walk 3	Yes/No<br>How long for?
Comments:<br>Tired		Comments:		Comments:	

Instructions:

For each day:

1. Please complete whether you took a walk Yes/No for Walk 1, Walk 2 and Walk 3
2. If you went for a walk, please enter the number of minutes the walk lasted
3. Please write any comments that you wish to about your experience of the walk or why you didn't go for a walk on this occasion

You do not have to walk three times a day. If you walk over three times a day, please enter the fourth (or fifth, sixth etc.) walk in a comments box for that day e.g. Walk 4: 15 minutes.

Example:

If you go for a walk twice today, once for 30 minutes and once for 10 minutes, you should write in the following:

Tuesday 26th January - Example					
Walk 1	Yes/No (Yes circled)<br>How long for?<br>30 min	Walk 2	Yes/No (Yes circled)<br>How long for?<br>10 min	Walk 3	Yes/No (No circled)<br>How long for?
Comments:		Comments:<br>Went to the shops		Comments:	

Please try and remember to complete the diary every day. Thank you for participating in this study.

Booklet Walking Diary

Name: _____________________ ID: _______

Week 4 January 2015

Monday 26th January					
Walk 1	Yes/No<br><br>How long for?	Walk 2	Yes/No<br><br>How long for?	Walk 3	Yes/No<br><br>How long for?
Comments:		Comments:		Comments:	
Tuesday 27th January					
Walk 1	Yes/No<br><br>How long for?	Walk 2	Yes/No<br><br>How long for?	Walk 3	Yes/No<br><br>How long for?
Comments:		Comments:		Comments:	
Wednesday 28th January					
Walk 1	Yes/No<br><br>How long for?	Walk 2	Yes/No<br><br>How long for?	Walk 3	Yes/No<br><br>How long for?
Comments:		Comments:		Comments:	

Thursday 29th January					
Walk 1	Yes/No<br><br>How long for?	Walk 2	Yes/No<br><br>How long for?	Walk 3	Yes/No<br><br>How long for?
Comments:		Comments:		Comments:	

Friday 30th January					
Walk 1	Yes/No<br><br>How long for?	Walk 2	Yes/No<br><br>How long for?	Walk 3	Yes/No<br><br>How long for?
Comments:		Comments:		Comments:	

Saturday 31st January					
Walk 1	Yes/No<br><br>How long for?	Walk 2	Yes/No<br><br>How long for?	Walk 3	Yes/No<br><br>How long for?
Comments:		Comments:		Comments:	

Sunday 1st February					
Walk 1	Yes/No<br><br>How long for?	Walk 2	Yes/No<br><br>How long for?	Walk 3	Yes/No<br><br>How long for?
Comments:		Comments:		Comments:	
