# Supplementary material for: Optimising the validity and completion of adherence diaries: a multiple case study and randomised crossover trial
Source: Trials. 2016 Oct 10;17:489. doi: 10.1186/s13063-016-1615-7 (PMC5057493; doi:10.1186/s13063-016-1615-7)
Supplement: Additional file 2: — EXACT: Acceptability Questionnaire. (PDF 273 kb) [file 13063_2016_1615_MOESM2_ESM.pdf]

EXACT: Acceptability Questionnaire

ID:________

Completing the Calendar Diary

We would like to know more about how you filled out your **Calendar Diary**. Please place an "X" on each line below at the point which matches your opinion. Please ensure your "X" cuts through the line.

Remembering to fill in the diary was ….

Very easy ____ Very hard

Remembering how often I had walked was ….

Very easy ____ Very hard

Completing the diary every day was….

No effort at all ____ A lot of effort

For me, completing the diary was…

Very useful ____ Not at all useful

Wearing the Activpal for a week was…

No effort at all ____ A lot of effort

For me, Activpal was…

Very useful ____ Not at all useful

I preferred using the…

Activpal ____ Calendar Diary

EXACT: Acceptability Questionnaire

ID:________

I completed my diary….

Please choose one option.

After every walk ☐

Once a day ☐

Every few days ☐

Once a week ☐

Less than once a week ☐

Each time it took me _______ minutes to complete the diary.

Do you have any other comments? (e.g. what you liked or disliked about the diary, whether it motivated you to walk)

THANK YOU FOR PARTICIPATING IN THIS STUDY AND COMPLETING THIS QUESTIONNAIRE

EXACT: Acceptability Questionnaire

ID:________

Completing the Booklet Diary

We would like to know more about how you filled out your **<u>Booklet Diary</u>**. Please place an "X" on each line below at the point which matches your opinion. Please ensure your "X" cuts through the line.

Remembering to fill in the diary was ….

Very easy ______________________________ Very hard

Remembering how often I had walked was ….

Very easy ______________________________ Very hard

Completing the diary every day was….

No effort at all ______________________________ A lot of effort

For me, completing the diary was…

Very useful ______________________________ Not at all useful

Wearing the Activpal for a week was…

No effort at all ______________________________ A lot of effort

For me, Activpal was…

Very useful ______________________________ Not at all useful

I preferred using the…

Activpal ______________________________ Booklet Diary

EXACT: Acceptability Questionnaire

ID:________

I preferred using the…

Calendar Diary ____ Booklet Diary

I completed my diary….

Please choose one option.

After every walk ☐

Once a day ☐

Every few days ☐

Once a week ☐

Less than once a week ☐

Each time it took me _______ minutes to complete the diary.

Do you have any other comments? (e.g. what you liked or disliked about the diary, whether it motivated you to walk)

THANK YOU FOR PARTICIPATING IN THIS STUDY AND COMPLETING THIS QUESTIONNAIRE
