# Supplementary material for: Optimising the validity and completion of adherence diaries: a multiple case study and randomised crossover trial
Source: Trials. 2016 Oct 10;17:489. doi: 10.1186/s13063-016-1615-7 (PMC5057493; doi:10.1186/s13063-016-1615-7)

**Additional file 3: Bland-Altman Plots for other analyses**

**Validity: Frequency adherence**

**Bland-Altman plot of the mean of the percentage walk frequency adherence as measured by optimised diary and Activpal vs the difference between the values**


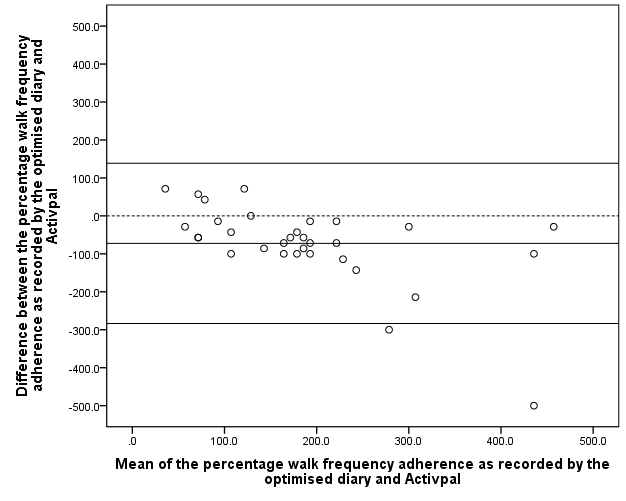


**Bland-Altman plot of the mean of the percentage walk frequency adherence as measured by non-optimised diary and Activpal vs the difference between the values**


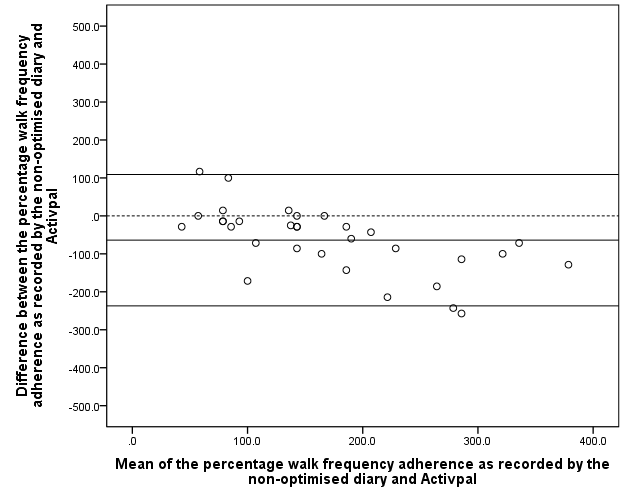


**Validity: Percentage of days adhered**

**Bland-Altman plot of the mean of the percentage of days adhered as recorded by the optimised diary and Activpal vs the difference between these values**


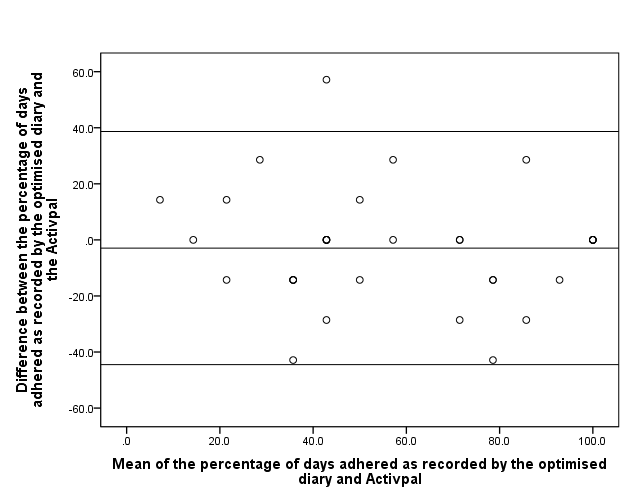


**Bland-Altman plot of the mean of the percentage of days adhered as recorded by the optimised diary and Activpal vs the difference between these values**


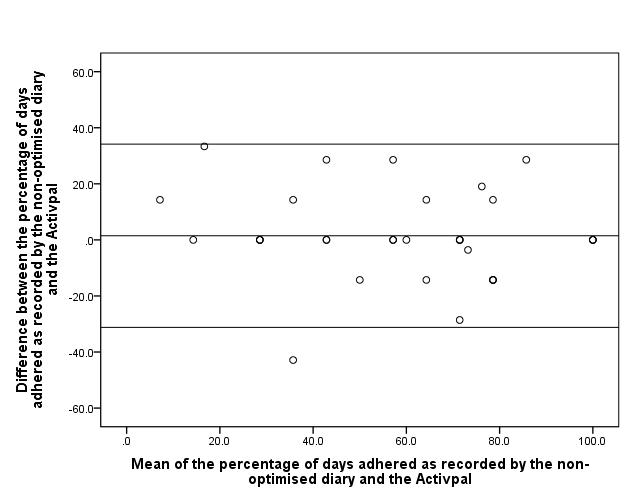


**Reliability: Duration adherence**

**Bland-Altman plot of the mean percentage walk duration adherence recorded in weeks 3 and 4 of the optimised diary vs the difference between these**


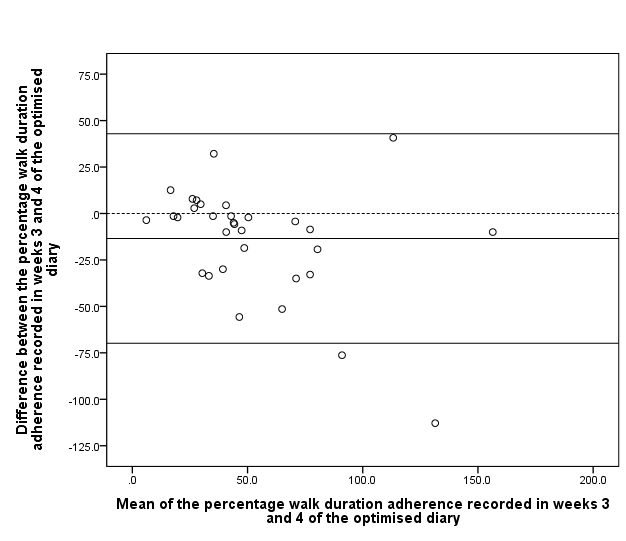


**Bland-Altman plot of the mean percentage walk duration adherence recorded in weeks 3 and 4 of the non-optimised diary vs the difference between these**


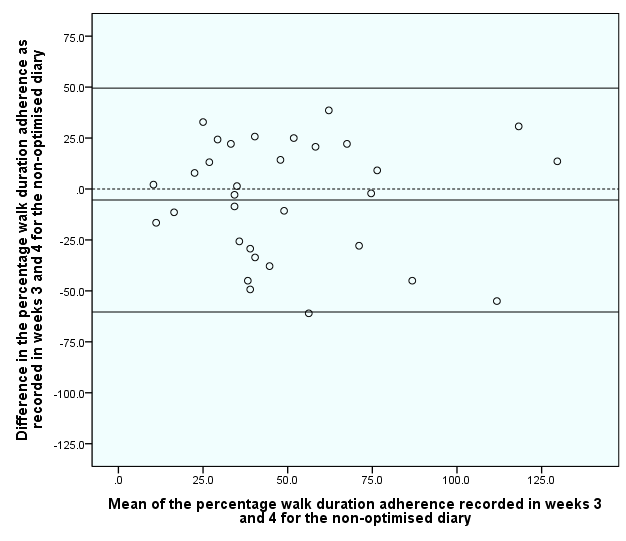


**Reliability: Frequency adherence**

**Bland-Altman plot of the mean of percentage walk frequency adherence as recorded in weeks 3 and 4 of the optimised diary vs the difference between them**


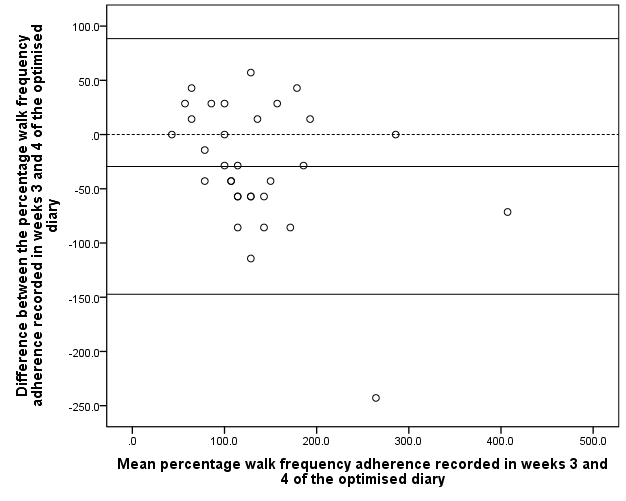


**Bland-Altman plot of the mean of percentage walk frequency adherence as recorded in weeks 3 and 4 of the non-optimised diary vs the difference between them**


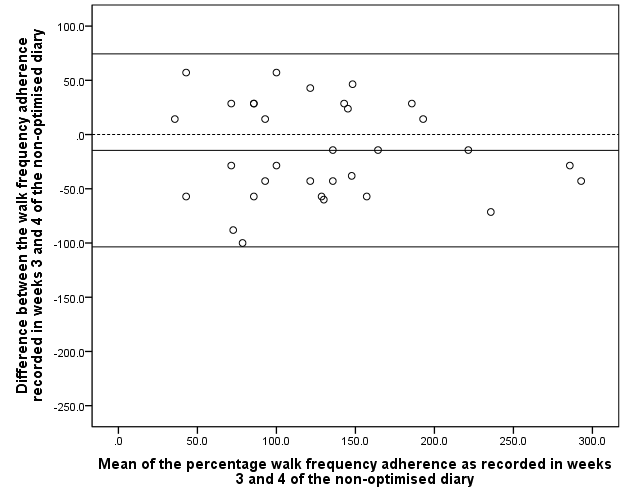


**Reliability: Percentage of days adhered**

**Bland-Altman plot of the mean of the percentage of days adhered as recorded in weeks 3 and 4 of the optimised diary vs the difference between these**


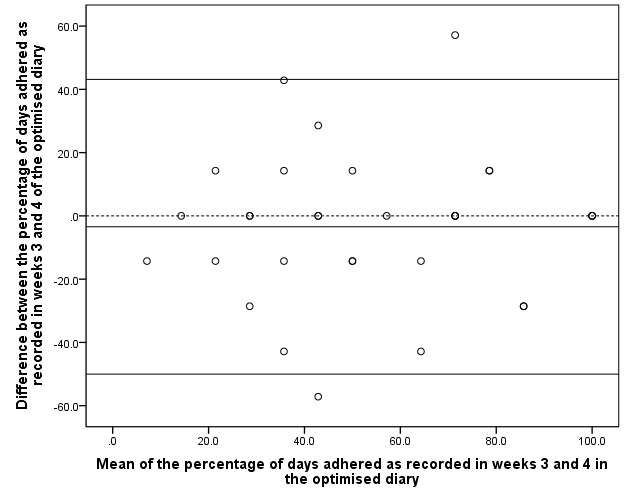


**Bland-Altman plot of the mean of the percentage of days adhered as recorded in weeks 3 and 4 of the non-optimised diary vs the difference between these**


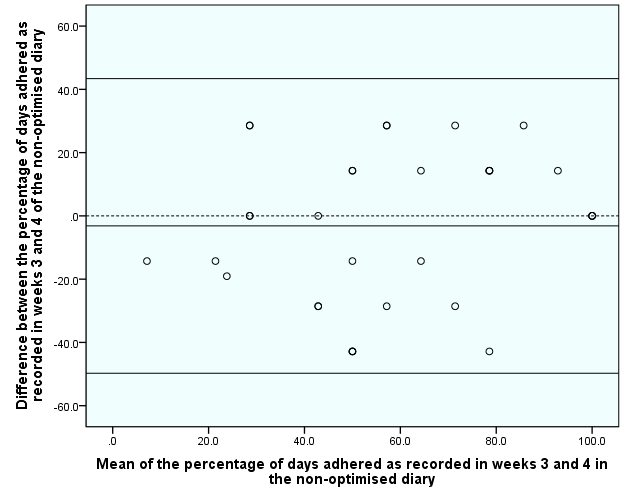

Supplement: Additional file 3: — Bland-Altman plots for other analyses. (DOCX 192 kb) [file 13063_2016_1615_MOESM3_ESM.docx]
